# Embryonic Neocortical Microglia Express Toll-Like Receptor 9 and Respond to Plasmid DNA Injected into the Ventricle: Technical Considerations Regarding Microglial Distribution in Electroporated Brain Walls

**DOI:** 10.1523/ENEURO.0312-18.2018

**Published:** 2018-11-29

**Authors:** Yuki Hattori, Takaki Miyata

**Affiliations:** Department of Anatomy and Cell Biology, Nagoya University Graduate School of Medicine, Nagoya 466-8550, Japan

**Keywords:** in utero electroporation, live-imaging, microglia, TLR4, TLR9, toll-like receptor

## Abstract

Microglia, the resident immune cells in the CNS, play multiple roles during development. In the embryonic cerebral wall, microglia modulate the functions of neural stem/progenitor cells through their distribution in regions undergoing cell proliferation and/or differentiation. Previous studies using CX3CR1-GFP transgenic mice demonstrated that microglia extensively survey these regions. To simultaneously visualize microglia and neural-lineage cells that interact with each other, we applied the *in utero* electroporation (IUE) technique, which has been widely used for gene-transfer in neurodevelopmental studies, to CX3CR1-GFP mice (males and females). However, we unexpectedly faced a technical problem: although microglia are normally distributed homogeneously throughout the mid-embryonic cortical wall with only limited luminal entry, the intraventricular presence of exogenously derived plasmid DNAs induced microglia to accumulate along the apical surface of the cortex and aggregate in the choroid plexus. This effect was independent of capillary needle puncture of the brain wall or application of electrical pulses. The microglial response occurred at plasmid DNA concentrations lower than those routinely used for IUE, and was mediated by activation of Toll-like receptor 9 (TLR9), an innate immune sensor that recognizes unmethylated cytosine-phosphate guanosine motifs abundant in microbial DNA. Administration of plasmid DNA together with oligonucleotide 2088, the antagonist of TLR9, partially restored the dispersed intramural localization of microglia and significantly decreased luminal accumulation of these cells. Thus, via TLR9, intraventricular plasmid DNA administration causes aberrant distribution of embryonic microglia, suggesting that the behavior of microglia in brain primordia subjected to IUE should be carefully interpreted.

## Significance Statement

Microglia have been recently shown to play multiple roles in the embryonic brain. In the trials for labeling neural-lineage cells using IUE technique in CX3CR1-GFP mice, in which microglia express GFP, to achieve dual live-imaging of these cell types, we unexpectedly found that intra-ventricular administration of plasmid DNA caused microglial aberrant accumulation along the luminal surface of the cerebral wall and in the choroid plexus. Notably, coadministration of TLR9 antagonist into the ventricle together with plasmid DNA significantly improved microglial localization in the mid-embryonic (E14) cortex, suggesting that massive microglial accumulation induced by plasmid DNA is primarily mediated by TLR9 activation. Our findings have implications for the application of IUE to investigate embryonic microglia.

## Introduction

Microglia, the resident macrophages of the CNS, are distributed throughout both adult and embryonic brain (Perry et al., 1985; Ashwell, 1991; Nimmerjahn et al., 2005; Monier et al., 2007; Swinnen et al., 2013). Embryonic microglia play multiple roles in development of neural-lineage cells, e.g., phagocytotically eliminating Tbr2^+^ intermediate progenitors (Cunningham et al., 2013; Barger et al., 2018), regulating the differentiation status of neural progenitor cells in the subventricular zone (SVZ) and ventricular zone (VZ; Arnò et al., 2014; Hattori and Miyata, 2018), and modulating cortical interneuron positioning (Squarzoni et al., 2014; Thion and Garel, 2017). Live-imaging studies of microglia using transgenic mice such as CX3CR1-GFP mice (Jung et al., 2000) have shown that microglia dynamically change their distribution during cortical development (Swinnen et al., 2013) and extensively survey proliferative zones in response to CXCL12 during the mid-embryonic period (Hattori and Miyata, 2018).

To further investigate how microglia and neural-lineage cells interact and/or collaborate (i.e., where, when, and for how long microglia contact undifferentiated and/or intermediate neural progenitors and whether these cell types mutually influence their development), it is necessary to simultaneously live-monitor microglia and neural lineage cells and observe them under genetic manipulation. For labeling and genetic modification of neural lineage cells of embryonic mammalian brains, the *in utero* electroporation (IUE) technique has been widely used (Fukuchi-Shimogori and Grove, 2001; Saito and Nakatsuji, 2001; Tabata and Nakajima, 2001). Because this technique is easily combined with the use of transgenic mice developed for visualization of certain cell types or subcellular structures (Okamoto et al., 2013; Shinoda et al., 2018), we predicted that it would be useful for monitoring microglia in CX3CR1-GFP mice. In pilot trials of this dual imaging approach (i.e., visualization of both microglia and non-microglia), however, we unexpectedly found that conventional IUE of the embryonic mouse cerebral wall markedly altered microglial distribution in the cortex. A recent study reported that IUE caused activation of embryonic microglia, and thus induced cell death, in the developing hypothalamus (Rosin and Kurrasch, 2018), but the underlying biological mechanisms remained unknown. In this study, we investigated the causes of abnormal microglial distribution and point to a potential molecular mechanism for this phenomenon.

## Materials and Methods

### Mice

CX3CR1-GFP mice (Jung et al., 2000; IMSR, Catalog #JAX:005582; RRID:IMSR_JAX:005582) were purchased from Jackson Laboratories. ICR mice were purchased from Japan SLC. Mice were housed under specific pathogen-free conditions at Nagoya University. All protocols for animal experiments were approved by the Institutional Animal Care and Use Committee of Nagoya University. To obtain CX3CR1-GFP^+^ embryos (heterozygous), male homozygous CX3CR1-GFP mice were mated with female ICR wild-type mice.

### Plasmid DNA and LPS injection into the lateral ventricle

**Figure 1.**
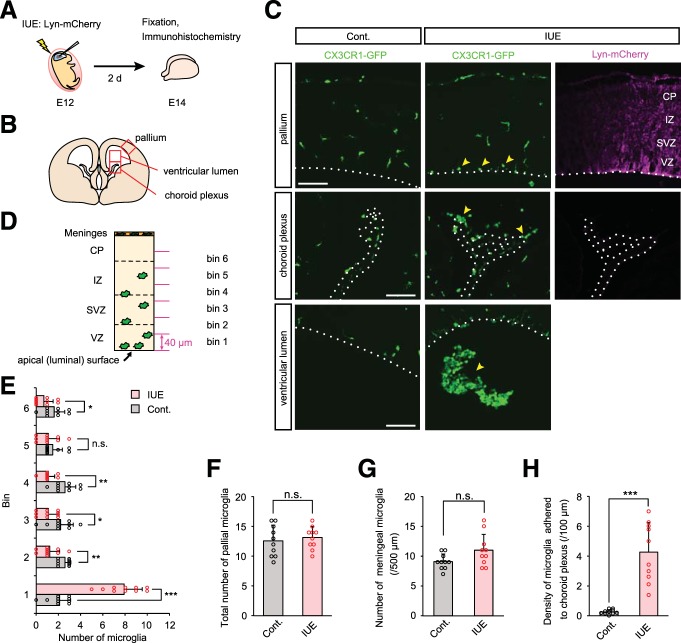
**IUE disturbs microglial distribution in the developing cerebral cortex. *A***, Experimental design of IUE. Plasmid DNA (pEFX2-Lyn-mCherry) was injected into the right lateral ventricle of an E12 CX3CR1-GFP mouse, and then electrical pulses were applied. After 2 d (E14), the brain was fixed and subjected to immunohistochemical analysis. ***B***, Illustration showing the approximate region of pallium, choroid plexus, and ventricular lumen for immunohistochemical analyses. ***C***, Representative immunostaining to detect GFP (CX3CR1) and RFP (Lyn-mCherry) in pallium, choroid plexus, and ventricular lumen of control and IUE brains. Broken lines show the apical surface of the pallium in the top and bottom, and the choroid plexus in the middle. Yellow arrowheads indicate microglia accumulated near the apical surface of the pallium, on the choroid plexus and in the ventricular lumen. Scale bar, 100 µm. ***D***, Bin definition for immunohistochemical analyses is shown. Each section in the cerebral wall was numbered from the ventricle side (bins 1–6, 40 µm each). ***E***–***G***, Graphs depicting numbers of microglia in each bin (40 µm × 300 µm square; ***E***), the total number within 240 µm from the apical surface of the pallium (***F***), and the number of meningeal microglia (***G***). ***H***, Density of microglia adhered to the choroid plexus in control versus IUE brains. For statistical analyses, *n* = 10 samples obtained from five embryos (2 sections, each) were quantified. One or two littermates per dam were subjected to a series of tests. Data represent mean ± SD. ***p < 0.001, **p < 0.01, *p < 0.05, or n.s., not significant; Mann–Whitney *U* test.

Plasmid DNA (pEFX2-Lyn-mCherry) purified using the QIAGEN Plasmid Maxi kit (catalog #12163, QIAGEN) or the EndoFree Plasmid Maxi kit (catalog #12362, QIAGEN) was dissolved in Tris-EDTA (10 mm Tris-HCl, 1 mm EDTA, pH 8.0) at a concentration of 5 µg/µl. The plasmid stock was diluted in saline solution to a concentration of 0.5 µg/µl. To monitor injection, Fast Green (0.1%) was added to the plasmid DNA solution at a ratio of 1:10. One microliter of plasmid DNA solution was injected into the lateral ventricle of the right hemisphere of embryonic day (E)12 mouse brain. The final concentration of plasmid DNA ranged 0.03–0.5 µg/µl, as indicated. After 2 d, the number and distribution pattern of microglia were quantified in the lateral part of the cerebral wall and choroid plexus (right hemisphere; Fig. 1*B*). LPS (Sigma-Aldrich) was diluted in saline solution to obtain a concentration of 2.5 ng, 250 pg, 25 pg, or 2.5 pg/µl and administered 1 µl of the solution into the lateral ventricle of E12 mouse brain. Regarding the amount of bacterial endotoxin contained in plasmid DNA solution, we referred to the manufacturer’s website (https://www.qiagen.com/us/resources/technologies/plasmid-resource-center/removal%20of%20bacterial%20endotoxins/).

### *In utero* electroporation

IUE was performed as described previously (Okamoto et al., 2013; Shinoda et al., 2018). After pregnant ICR mice were anesthetized by intraperitoneal injection of pentobarbital sodium (Somnopentyl; Kyoritsu Seiyaku), 1 µl of plasmid DNA solution was injected into the lateral ventricle of E12 mouse embryos. Briefly, the head of the embryo inside the uterus was placed between the disks of a forceps-type electrode (3 mm disk electrodes for E12; CUY650P3, NEPA GENE), and electric pulses (32 V) were applied four times, resulting in gene transfection into the cerebral wall.

### Administration of TLR9 antagonist together with plasmid DNA

Previous studies tested various oligonucleotides (ODNs) for their stimulatory or inhibitory activities for Toll-like receptor 9 (TLR9; Krieg et al., 1995; Stunz et al., 2002). Based on the finding that ODN 2088 is one of the most effective inhibitors, we applied it in our experiments as TLR9 antagonist. The ODN 2088 (5'-TCC TGG CGG GGA AGT-3') was purchased from Invivogen. The drug was suspended in endotoxin-free water and dissolved in plasmid DNA solution at a mass ratio of plasmid DNA/ODN 2088 1:1. Plasmid DNA and ODN 2088 were injected into the lateral ventricles of E12 embryos. After 2 d (E14), the brains were perfused with 4% PFA and subjected to immunohistochemistry.

### Immunohistochemistry

Immunohistochemistry was performed as described previously (Okamoto et al., 2013). Brains were fixed in 4% PFA, immersed in 20% sucrose, and frozen-sections (16 µm thick) were cut. Sections were treated with the following primary antibodies: rat anti-GFP (1:500, Nacalai Tesque, catalog #04404-84; RRID:AB_10013361) and rabbit anti-RFP (1:500, MBL, catalog #PM005; RRID:AB_591279). After washes, sections were treated with secondary antibodies conjugated to AlexaFluor 488 (1:400; Invitrogen, catalog #A-11006; RRID:AB_141373) or AlexaFluor 546 (1:400; Invitrogen, catalog #A-11010; RRID:AB_143156) and imaged on a BX60 fluorescence microscope (Olympus) or FV1000 confocal microscope (Olympus). The cerebral wall was divided into six bins (40 µm) and numbered in an inside-out fashion (bins 1–6). We counted the number of microglia of which somas were within the VZ (including ones along the apical surface) but excluded microglia whose somata were completely in the ventricular lumen although they partly attached to the apical surface.

### Cell sorting

Freshly isolated pallial walls derived from E14 male and female CX3CR1-GFP mice were treated with trypsin (0.05%, 3 min at 37°C). Dissociated pallial cells were filtered through a 40 μm strainer (Corning) to eliminate all remaining cell clumps, and then resuspended in DMEM containing 5% fetal bovine serum (Invitrogen), 5% horse serum (Invitrogen), and penicillin/streptomycin (50 U/ml, each; Meiji Seika Pharma). CX3CR1-GFP^+^ cells were sorted through a 100-μm nozzle by FACS Aria II (BD Biosciences). The drop delay was optimized using BD Biosciences Accudrop beads (BD Biosciences).

### Real-time PCR

First-strand cDNA was synthesized from ∼100 ng total RNA was reverse-transcribed into cDNA using SuperScript III reverse transcriptase (ThermoFisher Scientific) in the presence of RNase inhibitor (Thermo Fisher Scientific). Real-time PCR was performed with SYBR Green Real Time PCR Master (Toyobo) using Thermal Cycler Dice Real Time System TP800 (TaKaRa). To amplify specific transcripts, samples were heated at 95°C for 15 min and subsequently underwent a melting curve analysis from 60°C to 95°C. The threshold cycle number (*C*t) of the target was calculated and expressed relative to that of GAPDH, and then ΔΔ*C*t values of the target were calculated and presented as relative fold induction. Primers were: 5′-AGC CTC CGA GAC AAC TAC CT-3′ (sense) and 5′-TTG GTC AGG GCC TTT AGC TG-3′ (antisense) for *TLR9*; 5'-TCC CTG CAT AGA GGT AGT TCC TA-3' (sense) and 5'-TTC AAG GGG TTG AAG CTC AGA-3' (antisense) for *TLR4*; and 5'-GTT GTC TCC TGC GAC TTC A-3' (sense) and 5'-GGT GGT CCA GGG TTT CTT A-3' (antisense) for *GAPDH*.

### Live imaging in cortical slice culture

To obtain cortical slices covered with intact meninges, whole forebrains isolated from E14 male and female CX3CR1-GFP mice that had been electroporated at E12 were embedded in 2% agarose gel, and then sliced coronally (350 µm) using a vibratome. The slices were cultured in collagen gel as previously described (Miyata et al., 2004). Time-lapse imaging was performed on a CV1000 confocal microscope (Olympus). Chambers for on-stage culture were filled with 40% O_2_.

### Statistical analysis

Quantitative data are presented as mean ± SD from representative experiments. Statistical differences between groups were analyzed by Mann–Whitney *U* test for two-group comparisons or Steel–Dwass test for multiple comparisons using R software. *p* < 0.05 was considered significant. *p* values in every figure are separately listed in tables (Tables 1–7). Individual values were plotted as open circles in bar graphs. The number of samples examined in each analysis is shown in the figure legends.

## Results

### IUE disturbs microglial distribution in the developing cerebral cortex

To simultaneously visualize microglia and neural-lineage cells, we performed IUE on E12 CX3CR1-GFP mice (Jung et al., 2000). Briefly, plasmid DNA (pEFX2-Lyn-mCherry) was injected into the lateral ventricle of the right hemisphere of E12 mouse brain using a glass capillary needle, followed by electrical pulses across the embryo’s head (Fig. 1*A*). Surprisingly, immunohistochemical inspections 2 d later revealed that the distribution patterns of CX3CR1^+^ microglia in the pallium and choroid plexus were abnormal (Fig. 1*B*,*C*). Normally at E14, microglia are distributed diffusely throughout the pallium along the radial (ventricle-to-pia) axis, and are found in the VZ, SVZ, and intermediate zone (IZ; Perry et al., 1985; Ashwell, 1991; Monier et al., 2007; Cunningham et al., 2013; Swinnen et al., 2013). In brains subjected to IUE (hereafter, IUE brains), however, microglia were extremely scarce in both the SVZ and IZ (bins 2–4) and aberrantly accumulated along the apical surface (within 40 µm from the apical surface: bin 1; Fig. 1*C*–*E*; Table 1), with the total number of microglia in the pallium and meningeal microglia comparable between control (non-IUE) and IUE brains (Fig. 1*F*,*G*). IUE brains also exhibited densely accumulated microglia in the choroid plexus and the ventricle, whereas no such massive luminal infiltrations were observed in non-IUE controls (Fig. 1*C*,*H*). IUE caused the same type of aberrant microglial distribution in wild-type (ICR, non-CX3CR1-GFP transgenic) mice (data not shown). These results indicate that, in our hands, the standard IUE technique disturbed the localization of microglia in a manner suggestive of an attraction from the IZ or SVZ toward the ventricular lumen.

**Table 1.** Statistics for Figure 1

**Graph**	**Data structure**	**Type of test**	***p***
Fig. 1*E*	Nonparametric	Mann–Whitney *U* test	Bin 1: 1.1 × 10^−5^ Bin 2: 0.0022 Bin 3: 0.0307 Bin 4: 0.0014 Bin 5: 0.4281 Bin 6: 0.0495
Fig. 1*F*	Nonparametric	Mann–Whitney *U* test	0.6835
Fig. 1*G*	Nonparametric	Mann–Whitney *U* test	0.1021
Fig. 1*H*	Nonparametric	Mann–Whitney *U* test	1.1 × 10^−5^

### Plasmid DNA injection into the ventricle, without electrical pulses, results in abnormal microglial distribution

**Figure 2.**
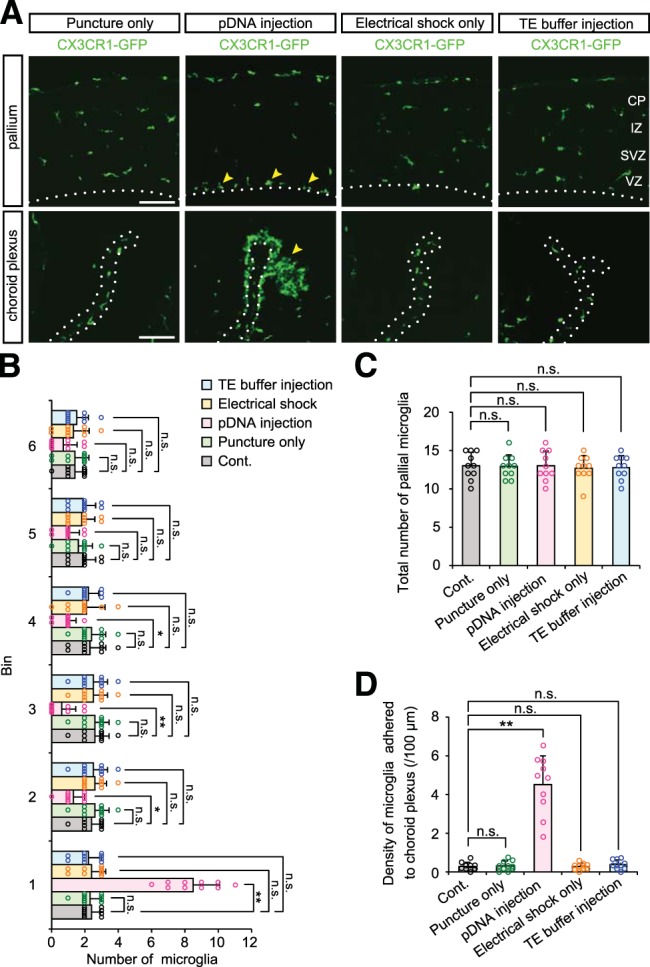
**Plasmid DNA injection into the ventricle, without electrical pulses, results in abnormal microglial distribution. *A***, Representative immunostaining for CX3CR1-GFP in the pallium and choroid plexus of mouse brains subjected to puncture with a glass capillary needle, injection of plasmid DNA (shown as pDNA) into the lateral ventricle, electrical pulses, or injection of Tris-EDTA solution (10 mm Tris-HCl, 1 mm EDTA, pH 8.0) alone without plasmid DNA. Yellow arrowheads indicate microglia accumulated near the apical surface of the cerebral wall or adhered to the choroid plexus. Broken lines show the apical surface of the pallium in the top and the choroid plexus in the bottom. Scale bar, 100 µm. ***B***, ***C***, Graphs depicting the number of microglia positioned in each 40 µm bin (***B***) and the total number of these cells within 240 µm from the apical surface (***C***) in brains that were subjected to each procedure. ***D***, Density of microglia adhered to the choroid plexus. For statistical analyses, *n* = 10 samples obtained from five embryos (2 sections, each) were quantified. One or two littermates per dam were subjected to a series of tests. Data represent mean ± SD. ***p < 0.001, **p < 0.01, *p < 0.05, or n.s., not significant; Steel–Dwass test.

To determine which of the steps involved in IUE (1, puncturing the cerebral wall with a glass capillary needle; 2, injection of plasmid vector DNA into the lateral ventricle; 3, electrical pulses) causes microglial aberrant accumulation, we compared the distribution of microglia between embryos subjected to each of these procedures separately. When the cerebral wall was only punctured with a glass capillary needle, but no solution was injected, microglia were still distributed homogenously throughout the cortex, as in control (nontreated) brains (Fig. 2*A*–*D*; Table 2). By contrast, brains that were intraventricularly injected with plasmid DNA (pEFX2-Lyn-mCherry) but not subjected to electrical pulses exhibited massive microglial accumulation near the ventricle in the VZ and their infiltration in the choroid plexus. On the other hand, electrical pulses alone did not result in aberrant microglial distribution. In another control group injected with Tris-EDTA solution (10 mm Tris-HCl, 1 mm EDTA, pH 8.0) alone, microglia showed normal distribution pattern in the cerebral wall and did not aggregate in the choroid plexus (Fig. 2*A*–*D*). These results strongly suggest that the presence of exogenously sourced plasmid DNAs in embryonic mouse ventricle caused abnormal microglial distribution.

**Table 2.** Statistics for Figure 2

**Graph**	**Data structure**	**Type of test**	***p***
Fig. 2*B*, bin 1	Nonparametric	Steel–Dwass	Cont vs Puncture only, *p* = 0.9990; Cont vs pDNA injection, *p* = 0.0011; Cont vs Electrical shock only, *p* = 0.9973; Cont vs TE buffer injection, *p* = 0.9871
Fig. 2*B*, bin 2	Nonparametric	Steel–Dwass	Cont vs Puncture only, *p* = 0.9781; Cont vs pDNA injection, *p* = 0.0369; Cont vs Electrical shock only, *p* = 0.9937; Cont vs TE buffer injection, *p* = 0.9996
Fig. 2*B*, bin 3	Nonparametric	Steel–Dwass	Cont vs Puncture only, *p* = 1.0000; Cont vs pDNA injection, *p* = 0.0055; Cont vs Electrical shock only, *p* = 0.9976; Cont vs TE buffer injection, *p* = 0.9976
Fig. 2*B*, bin 4	Nonparametric	Steel–Dwass	Cont vs Puncture only, *p* = 0.9992; Cont vs pDNA injection, *p* = 0.0154; Cont vs Electrical shock only, *p* = 0.9964; Cont vs TE buffer injection, *p* = 0.9996
Fig. 2*B*, bin 5	Nonparametric	Steel–Dwass	Cont vs Puncture only, *p* = 0.9473; Cont vs pDNA injection, *p* = 0.1056; Cont vs Electrical shock only, *p* = 0.9976; Cont vs TE buffer injection, *p* = 1.0000
Fig. 2*B*, bin 6	Nonparametric	Steel–Dwass	Cont vs Puncture only, *p* = 0.9998; Cont vs pDNA injection, *p* = 0.3261; Cont vs Electrical shock only, *p* = 0.9962; Cont vs TE buffer injection, *p* = 1.0000
Fig. 2*C*	Nonparametric	Steel–Dwass	Cont vs Puncture only, *p* = 0.9994; Cont vs pDNA injection, *p* = 1.0000; Cont vs Electrical shock only, *p* = 0.9969; Cont vs TE buffer injection, *p* = 0.9981
Fig. 2*D*	Nonparametric	Steel–Dwass	Cont vs Puncture only, *p* = 0.9989; Cont vs pDNA injection, *p* = 0.0015; Cont vs Electrical shock only, *p* = 0.9994; Cont vs TE buffer injection, *p* = 0.9913

### Timing and sensitivity of microglial response to intraventricularly injected plasmid DNAs

**Figure 3.**
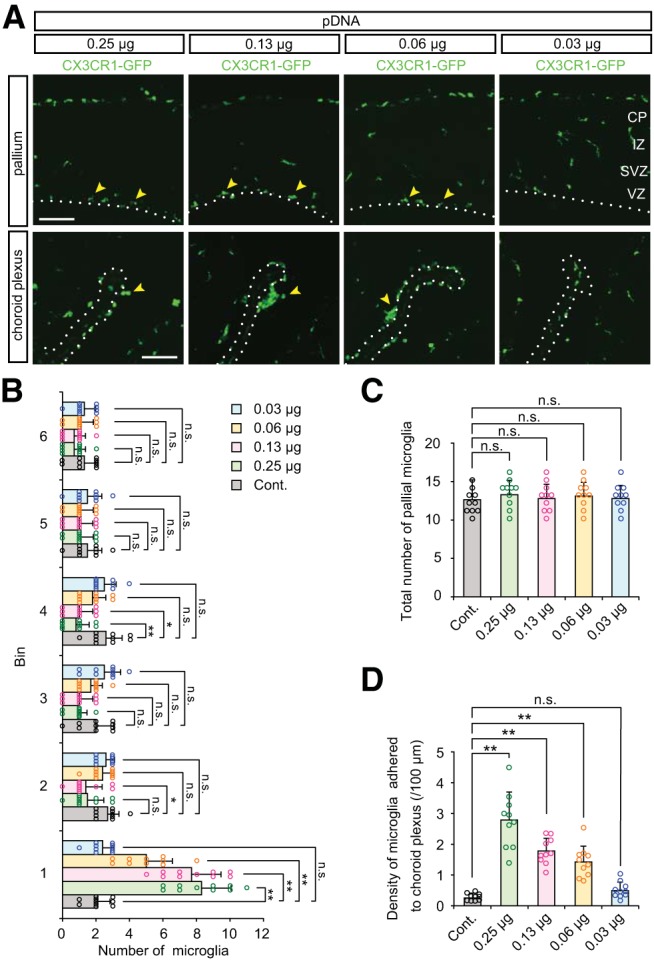
**Sensitivity of microglial response to intraventricularly injected plasmid DNAs. *A***, CX3CR1-GFP immunostaining showing microglial accumulation in brains injected with the indicated amount of plasmid DNA (0.25, 0.13, 0.06, and 0.03 µg). Yellow arrowheads indicate microglia accumulated near the apical surface of the pallium and on the choroid plexus. Scale bar, 100 µm. ***B***, ***C***, Graphs depicting the number of microglia positioned in each 40 µm bin (***B***) and the total number of these cells within 240 µm from the apical surface (***C***) in brains that were injected with plasmid DNA. ***D***, Density of microglia adhered to the choroid plexus. For statistical analyses, *n* = 10 samples obtained from five embryos (2 sections, each) were quantified. One or two littermates per dam were subjected to a series of tests. Data represent mean ± SD. ***p < 0.001, **p < 0.01, *p < 0.05, or n.s., not significant; Steel–Dwass test.

Next, we sought to determine the sensitivity of intramural microglia to intraventricular plasmid DNAs. To compare the threshold amount of DNA required to provoke microglial responses with the amounts of DNA used in standard IUE protocols (0.5–1.0 µg per unilateral ventricular space; Okamoto et al., 2013; Shinoda et al., 2018), we injected solutions containing various amounts of pEFX2-Lyn-mCherry (0.25, 0.13, 0.06, and 0.03 µg) into the lateral ventricles of E12 embryos. After 2 d (E14), microglial accumulation near the ventricle was still observed in brains injected with 0.25, 0.13, or 0.06 µg plasmid DNA (Fig. 3*A*,*B*; Table 3), with no increase of the total number of pallial microglia (Fig. 3*C*). We also found dose-dependent accumulation of microglia in the choroid plexus (Fig. 3*D*). By contrast, in brains injected with 0.03 µg plasmid DNA, microglia were observed in a normal pattern (widely distributed from the VZ to IZ) with no accumulation in the choroid plexus. These results showed that amounts of plasmid DNA much smaller than those conventionally used for IUE can cause microglia to infiltrate toward and in the ventricular lumen.

**Table 3.** Statistics for Figure 3

**Graph**	**Data structure**	**Type of test**	***p***
Fig. 3*B*, bin 1	Nonparametric	Steel–Dwass	Cont vs 0.25 µg, *p* = 0.0013; Cont vs 0.13 µg, *p* = 0.0013; Cont vs 0.06 µg, *p* = 0.0028; Cont vs 0.03 µg, *p* = 0.8787
Fig. 3*B*, bin 2	Nonparametric	Steel–Dwass	Cont vs 0.25 µg, *p* = 0.0693; Cont vs 0.13 µg, *p* = 0.0453; Cont vs 0.06 µg, *p* = 0.9317; Cont vs 0.03 µg, *p* = 0.9990
Fig. 3*B*, bin 3	Nonparametric	Steel–Dwass	Cont vs 0.25 µg, *p* = 0.1044; Cont vs 0.13 µg, *p* = 0.2141; Cont vs 0.06 µg, *p* = 0.8898; Cont vs 0.03 µg, *p* = 0.8352
Fig. 3*B*, bin 4	Nonparametric	Steel–Dwass	Cont vs 0.25 µg, *p* = 0.0098; Cont vs 0.13 µg, *p* = 0.0196; Cont vs 0.06 µg, *p* = 0.3581; Cont vs 0.03 µg, *p* = 0.9985
Fig. 3*B*, bin 5	Nonparametric	Steel–Dwass	Cont vs 0.25 µg, *p* = 0.8286; Cont vs 0.13 µg, *p* = 0.7255; Cont vs 0.06 µg, *p* = 0.8286; Cont vs 0.03 µg, *p* = 1.0000
Fig. 3*B*, bin 6	Nonparametric	Steel–Dwass	Cont vs 0.25 µg, *p* = 0.4412; Cont vs 0.13 µg, *p* = 0.4412; Cont vs 0.06 µg, *p* = 0.9667; Cont vs 0.03 µg, *p* = 0.9999
Fig. 3*C*	Nonparametric	Steel–Dwass	Cont vs 0.25 µg, *p* = 0.6147; Cont vs 0.13 µg, *p* = 0.9493; Cont vs 0.06 µg, *p* = 0.7574; Cont vs 0.03 µg, *p* = 0.9162
Fig. 3*D*	Nonparametric	Steel–Dwass	Cont vs 0.25 µg, *p* = 0.0015; Cont vs 0.13 µg, *p* = 0.0015; Cont vs 0.06 µg, *p* = 0.0015; Cont vs 0.03 µg, *p* = 0.1825

**Figure 4.**
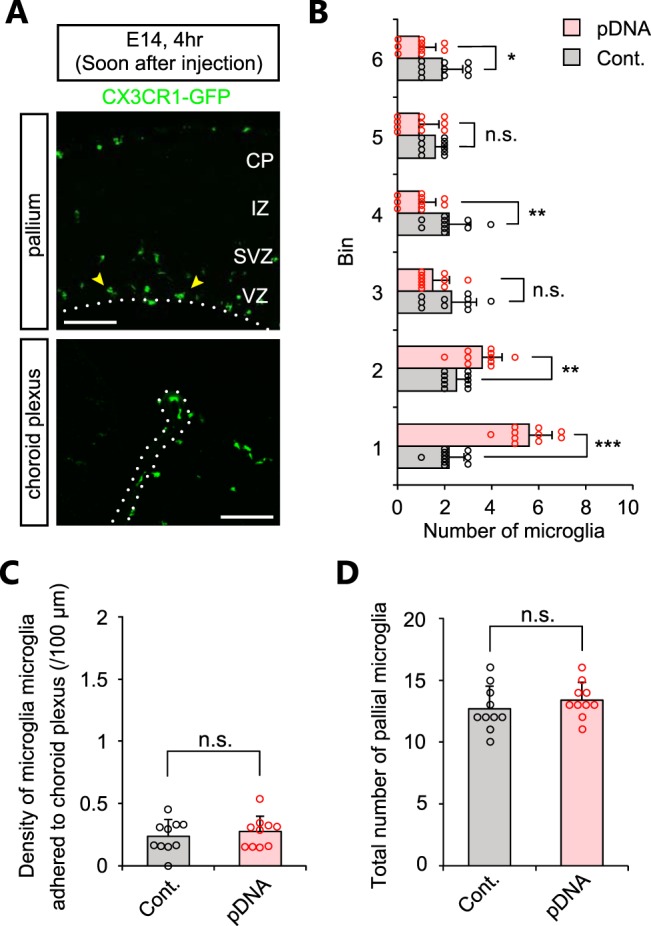
**Microglia immediately sense plasmid DNAs injected into the ventricle. *A***, Representative immunostaining of CX3CR1-GFP in E14 brain fixed soon (4 h) after administration of 0.5 µg plasmid DNA. Yellow arrowheads show microglia accumulated near the apical surface of the pallium and on the choroid plexus. Scale bar, 100 µm. ***B***, Graph showing the number of pallial microglia positioned in each 40 µm bin in control and plasmid-injected brains. ***C***, Graph comparing density of microglia adhered to choroid plexus. ***D***, The total number of pallial microglia within 240 µm from the apical surface. For statistical analyses, *n* = 10 samples obtained from five embryos (2 sections, each) were quantified. Two or three littermates per dam were subjected to a series *of* tests. Data represent mean ± SD. ***p < 0.001, **p < 0.01, *p < 0.05, or n.s., not significant; Mann–Whitney *U* test.

To determine how quickly microglia infiltrate into the DNA-injected lumen, we analyzed E14 brains soon (4 h) after administration of plasmid DNA solution (0.5 µg plasmid DNA), and found that the distribution of microglia was already abnormal. Specifically, microglia had departed from their original locations (the IZ, SVZ, and upper VZ) toward the apical surface (Fig. 4*A*,*B*; Table 4), although they had not yet accumulated in the choroid plexus (Fig. 4*A*,*C*). The total number of pallial microglia was comparable between plasmid DNA-treated and control brains (Fig. 4*D*). These results suggest that intramural microglia can immediately sense plasmid DNAs injected into the ventricle, leading to a change in their regional distribution.

**Table 4:** Statistics for Figure 4

**Graph**	**Data structure**	**Type of test**	***p***
Fig. 4*B*	Nonparametric	Mann–Whitney *U*	bin 1: 1.1 × 10^−5^ bin 2: 0.0074 bin 3: 0.1023 bin 4: 0.0058 bin 5: 0.0837 bin 6: 0.0275
Fig. 4*C*	Nonparametric	Mann–Whitney *U*	0.9869
Fig. 4*D*	Nonparametric	Mann–Whitney *U*	0.2789

### Intraventricular administration of TLR9 antagonist decreases microglial infiltration induced by plasmid DNA injection

Macrophages, including microglia, express TLRs, prototype pattern-recognition receptors (PRRs) that recognize pathogen-associated molecular patterns (PAMPs) from microorganisms and thus initiate innate immune responses after viral or bacterial infection (Akira and Takeda, 2004; Takeuchi and Akira, 2010; O’Neill et al., 2013; Vijay, 2018). Among these receptors, TLR9 recognizes unmethylated CpG motifs, which are characteristic of bacterial and viral DNAs (Krieg et al., 1995; Hemmi et al., 2000; Bauer et al., 2001; Kumagai et al., 2008). TLR9 is expressed in microglia in the postnatal and adult brain (Doi et al., 2009; Butchi et al., 2011; Christensen et al., 2014; Matsuda et al., 2015; Cho and Hsieh, 2016; Scholtzova et al., 2017). Within cells, TLR9 primarily resides in the intracellular compartment (i.e., late-endosome/lysosome) and binds to CpG motifs after internalization of microbial DNA (Takeshita et al., 2001; Ahmad-Nejad et al., 2002; Barton et al., 2006; Chockalingam et al., 2009). Hence, we investigated whether plasmid DNA (usually produced in *Escherichia coli*) might evoke innate immune responses in microglia via TLR9.

**Figure 5.**
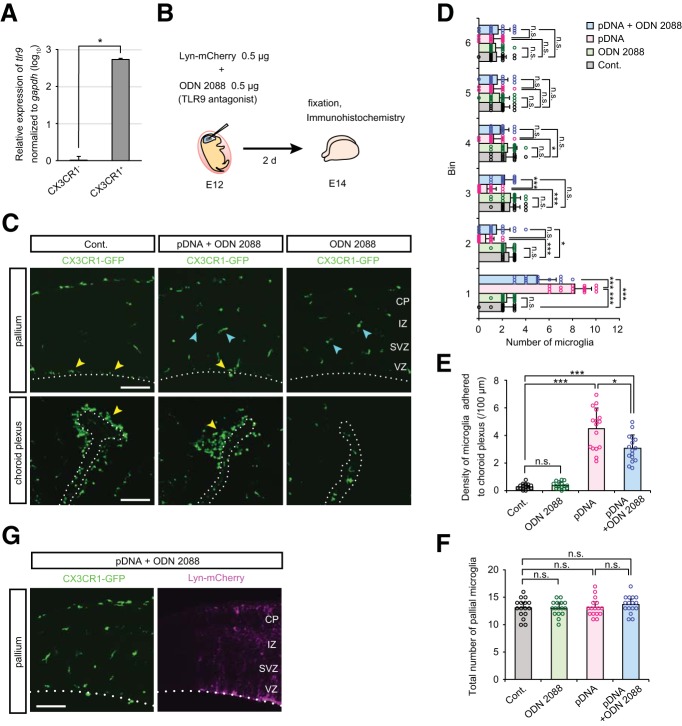
**Intraventricular administration of TLR9 antagonist decreases microglial infiltration induced by plasmid DNA injection. *A***, Relative expression of *TLR9* (normalized against *GAPDH*) in FACS-isolated CX3CR1^-^ and CX3CR1^+^ cells derived from the cerebral wall of E14 CX3CR1-GFP mice. Data represent mean ± SD (*n* = 4 samples obtained from independent experiments; *p* = 0.0286, Mann–Whitney *U* test). ***B***, Experimental design for ODN 2088 treatment. ODN 2088 was injected together with plasmid DNA into the lateral ventricle of E12 CX3CR1-GFP mice, and after 2 d (E14) the embryonic brains were fixed. ***C***, Immunofluorescence with anti-GFP antibody, showing the distribution of microglia in the pallium and choroid plexus. Yellow arrowheads indicate microglia aberrantly accumulated on the apical surface of the pallium or in the choroid plexus. Cyan arrowheads show microglia which were almost homogenously distributed in the cerebral wall. Scale bar, 100 µm. ***D***, ***E***, Graphs indicate the number of CX3CR1-GFP^+^ cells in each 40 µm bin of the pallium (***D***) and density of microglia directly adhered to the choroid plexus (***E***), comparing control, only ODN 2088-treated, plasmid DNA-injected, and plasmid DNA + ODN 2088 coinjected brains. ***F***, Graph showing the total number of pallial microglia within 240 µm from the apical surface. ***G***, Double-immunofluorescence for GFP (CX3CR1) and RFP (Lyn-mCherry) in the cortex of IUE E14 brain treated with ODN 2088. Microglia exhibited a normal distribution pattern in the Lyn-mCherry expressing region where IUE succeeded (Movies 1). Scale bar, 100 µm. For statistical analyses in ***D***–***F***, *n* = 16 samples obtained from eight embryos (2 sections, each) were quantified. Two or three littermates per dam were subjected to a series *of* tests. Data represent mean ± SD. ***p < 0.001, **p < 0.01, *p < 0.05, or n.s., not significant; Steel–Dwass test.

To determine whether embryonic microglia express TLR9, we performed real-time quantitative PCR on CX3CR1-GFP^+^ microglia and CX3CR1-GFP^-^ cells (most of which are of the neural lineage) isolated by cell sorting from the cortical wall of E14 CX3CR1-GFP mice. CX3CR1^+^ microglia expressed 529-fold higher level of TLR9 compared with CX3CR1^−^ cells (*p* = 0.0286, Mann–Whitney *U* test; Fig. 5*A*).

Movie 1. Live-imaging of microglia in plasmid DNA-treated brains. Live imaging of microglia in a cortical slice derived from a CX3CR1-GFP mouse brain transfected with Lyn-mCherry. Time-lapse imaging covers a period of 10 h (1 image/10 min). Green, CX3CR1-GFP; magenta, Lyn-mCherry. Scale bar, 100 µm. 10.1523/ENEURO.0312-18.2018.video.1

Next, to investigate whether microglial accumulation caused by plasmid DNA administration was mediated by TLR9, we coinjected ODN 2088, an inhibitory oligonucleotide that acts as a TLR9 antagonist (Stunz et al., 2002), into the mouse ventricle along with plasmid DNA (0.5 µg; Fig. 5*B*). ODN 2088 treatment partially restored the number of microglia localized in the SVZ/IZ and significantly reduced their accumulation along the apical surface, although it did not entirely rescue abnormal distribution [the number of microglia in bin 1 was still higher than control (nontreated) or only ODN 2088-treated brains; Fig. 5*C*,*D*; Table 5]. In addition, microglial infiltration in the choroid plexus was significantly reduced in ODN 2088-treated brains but still greater than control groups (Fig. 5*E*). On the other hand, the total number of microglia in the cortex was comparable between brains injected with plasmid DNA alone and those coinjected with plasmid DNA and ODN 2088 (Fig. 5*F*). Together, these results suggest that microglia expressing TLR9 may sense intraventricularly injected plasmid DNA and subsequently accumulate near the apical surface in the VZ and in the choroid plexus. Furthermore, we confirmed that performing IUE with Lyn-mCherry vector in the presence of ODN 2088 enabled us to prepare fresh slice cultures in which CX3CR1-GFP^+^ microglia were almost normally distributed and neural-lineage cells were labeled red (Fig. 5*G*; Movies 1 and 2).

**Table 5:** Statistics for Figure 5

**Graph**	**Data structure**	**Type of test**	***p***
Fig. 5*D*, bin 1	Nonparametric	Steel–Dwass	Cont vs ODN 2088, *p* = 0.8335; Cont vs pDNA, *p* = 5.6 × 10^−6^; pDNA vs. pDNA + ODN 2088, *p* = 2.4 × 10^−4^; Cont vs pDNA + ODN 2088, *p* = 2.1 × 10^−5^
Fig. 5*D*, bin 2	Nonparametric	Steel–Dwass	Cont vs ODN 2088, *p* = 0.6401; Cont vs pDNA, *p* = 1.3 × 10^−5^; pDNA vs. pDNA + ODN 2088, *p* = 0.0627; Cont vs pDNA + ODN 2088, *p* = 0.0163
Fig. 5*D*, bin 3	Nonparametric	Steel–Dwass	Cont vs ODN 2088, *p* = 0.9781; Cont vs pDNA, *p* = 8.2 × 10^−5^; pDNA vs. pDNA + ODN 2088, *p* = 4.7 × 10^−4^; Cont vs pDNA + ODN 2088, *p* = 0.5691
Fig. 5*D*, bin 4	Nonparametric	Steel–Dwass	Cont vs ODN 2088, *p* = 0.9746; Cont vs pDNA, *p* = 0.0102; pDNA vs. pDNA + ODN 2088, *p* = 0.1967; Cont vs pDNA + ODN 2088, *p* = 0.3568
Fig. 5*D*, bin 5	Nonparametric	Steel–Dwass	Cont vs ODN 2088, *p* = 0.9276; Cont vs pDNA, *p* = 0.0610; pDNA vs. pDNA + ODN 2088, *p* = 0.7661; Cont vs pDNA + ODN 2088, *p* = 0.5053
Fig. 5*D*, bin 6	Nonparametric	Steel–Dwass	Cont vs ODN 2088, *p* = 0.9955; Cont vs pDNA, *p* = 0.9158; pDNA vs. pDNA + ODN 2088, *p* = 0.7539; Cont vs pDNA + ODN 2088, *p* = 0.9840
Fig. 5*E*	Nonparametric	Steel–Dwass	Cont vs ODN 2088, *p* = 0.8121; Cont vs pDNA, *p* = 8.4 × 10^−6^; pDNA vs. pDNA + ODN 2088, *p* = 0.0374; Cont vs pDNA + ODN 2088, *p* = 8.4 × 10^−6^
Fig. 5*F*	Nonparametric	Steel–Dwass	Cont vs ODN 2088, *p* = 0.9966; Cont vs pDNA, *p* = 0.9982; pDNA vs. pDNA + ODN 2088, *p* = 0.6688; Cont vs pDNA + ODN 2088, *p* = 0.7716

Movie 2. Live-imaging of microglia in plasmid DNA and ODN 2088 coinjected brains. Live imaging of microglia in a cortical slice derived from a CX3CR1-GFP mouse brain transfected Lyn-mCherry with coadministration of ODN 2088. Time-lapse imaging covers a period of 10 h (1 image/10 min). Green, CX3CR1-GFP; magenta, Lyn-mCherry. Scale bar, 100 µm. 10.1523/ENEURO.0312-18.2018.video.2

### Endotoxins, if contained in plasmid DNA solution, trigger microglial aberrant accumulation

**Figure 6.**
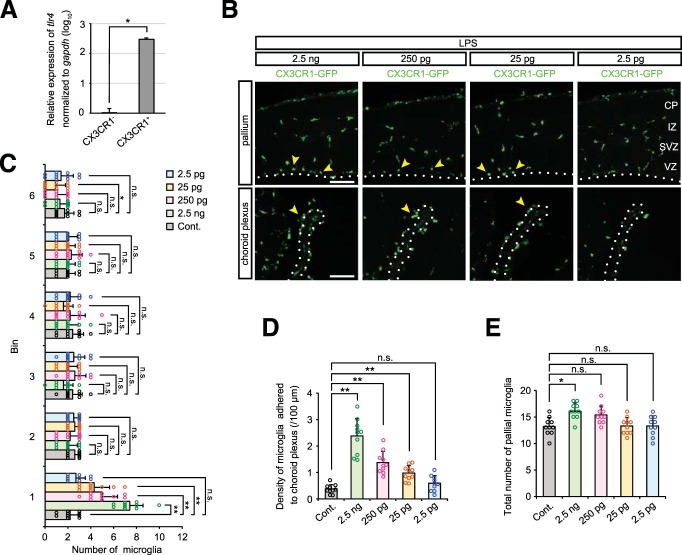
**Endotoxins trigger microglial aberrant accumulation. *A***, Relative expression of *TLR4* (normalized against *GAPDH*) in FACS-isolated CX3CR1^−^ and CX3CR1^+^ cells derived from the cerebral wall of E14 CX3CR1-GFP mice. Data represent mean ± SD (*n* = 4 samples obtained from independent experiments; *p* = 0.0286, Mann–Whitney *U* test). ***B***, Immunofluorescence with anti-GFP antibody, showing the distribution of microglia in the pallium and choroid plexus in brains injected with the indicated amount of LPS (2.5 ng, 250 pg, 25 pg, and 2.5 pg). Yellow arrowheads indicate microglia accumulated near the apical surface of the pallium and on the choroid plexus. Scale bar, 100 µm. ***C***, ***D***, Graphs depicting the number of pallial microglia positioned in each bin (***C***) and density of microglia adhered to the choroid plexus (***D***) in brains treated with various amounts of LPS. ***E***, The total number of pallial microglia within 240 µm from the apical surface. For statistical analyses in ***C***–***E***, *n* = 10 samples obtained from five embryos (2 sections, each) were quantified. One or Two littermates per dam were subjected to a series of tests. Data represent mean ± SD. ***p < 0.001, **p < 0.01, *p < 0.05, or n.s., not significant; Steel–Dwass test.

Although ODN 2088 treatment partially improved microglial distribution in the embryonic brain, microglia still accumulated near the apical surface of the cerebral wall. We postulated that the presence of bacterial endotoxin, lipopolysaccharide (LPS), in plasmid preparations might influence embryonic microglia. Because CX3CR1^+^ microglia derived from E14 cerebral wall expressed TLR4, a receptor for LPS (Akira and Takeda, 2004), much higher (290-fold higher level) than CX3CR1^−^ neural lineage cells (*p* = 0.0286, Mann–Whitney *U* test; Fig. 6*A*), we wanted to test whether LPS might elicit microglial activation in a separate manner from TLR9 signaling, and also determine how much LPS would be required to cause microglial abnormal localization.

Our routine preparations of plasmid (QIAGEN Plasmid Maxi Kit) for IUE yields relatively pure DNA with low levels of endotoxin [9.3 endotoxin unit (EU)/µg plasmid DNA; typically, 1 ng LPS corresponds to 1–10 EU, e.g., 0.47–4.7 ng LPS is estimated to be contained per 0.5 μg plasmid DNA]. When LPS alone diluted in saline was injected into the lateral ventricles of E12 embryos, immunohistochemistry after 2 d (at E14) demonstrated that, in brains treated with 2.5 ng, 250 pg, and 25 pg LPS, microglia were abnormally distributed (Fig. 6*B*–*D*; Table 6), which was coupled with an increase of the total number of pallial microglia in 2.5 ng LPS-treated cases (Fig. 6*E*). On the other hand, microglia showed normal localization in brains exposed to 2.5 pg LPS. This indicates that much lower levels of LPS than that contained in plasmid DNA solution to be used for IUE may substantially trigger microglial response.

**Table 6:** Statistics for Figure 6

**Graph**	**Data structure**	**Type of test**	***p***
Fig. 6*C*, bin 1	Nonparametric	Steel–Dwass	Cont vs 2.5 ng, *p* = 0.0012; Cont vs 250 pg, *p* = 0.0021; Cont vs 25 pg, *p* = 0.0055; Cont vs 2.5 pg, *p* = 0.7868
Fig. 6*C*, bin 2	Nonparametric	Steel–Dwass	Cont vs 2.5 ng, *p* = 0.4164; Cont vs 250 pg, *p* = 0.8867; Cont vs 25 pg, *p* = 1.0000; Cont vs 2.5 pg, *p* = 0.9924
Fig. 6*C*, bin 3	Nonparametric	Steel–Dwass	Cont vs 2.5 ng, *p* = 0.3094; Cont vs 250 pg, *p* = 0.9889; Cont vs 25 pg, *p* = 0.8691; Cont vs 2.5 pg, *p* = 0.9998
Fig. 6*C*, bin 4	Nonparametric	Steel–Dwass	Cont vs 2.5 ng, *p* = 0.7372; Cont vs 250 pg, *p* = 0.9700; Cont vs 25 pg, *p* = 0.3816; Cont vs 2.5 pg, *p* = 0.9811
Fig. 6*C*, bin 5	Nonparametric	Steel–Dwass	Cont vs 2.5 ng, *p* = 1.0000; Cont vs 250 pg, *p* = 0.8700; Cont vs 25 pg, *p* = 1.0000; Cont vs 2.5 pg, *p* = 0.9986
Fig. 6*C*, bin 6	Nonparametric	Steel–Dwass	Cont vs 2.5 ng, *p* = 0.9278; Cont vs 250 pg, *p* = 0.6779; Cont vs 25 pg, *p* = 0.3559; Cont vs 2.5 pg, *p* = 0.9514
Fig. 6*D*	Nonparametric	Steel–Dwass	Cont vs 2.5 ng, *p* = 0.0015; Cont vs 250 pg, *p* = 0.0015; Cont vs 25 pg, *p* = 0.0026; Cont vs 2.5 pg, *p* = 0.5054
Fig. 6*E*	Nonparametric	Steel–Dwass	Cont vs 2.5 ng, *p* = 0.0190; Cont vs 250 pg, *p* = 0.0845; Cont vs 25 pg, *p* = 1.0000; Cont vs 2.5 pg, *p* = 0.9994

**Figure 7.**
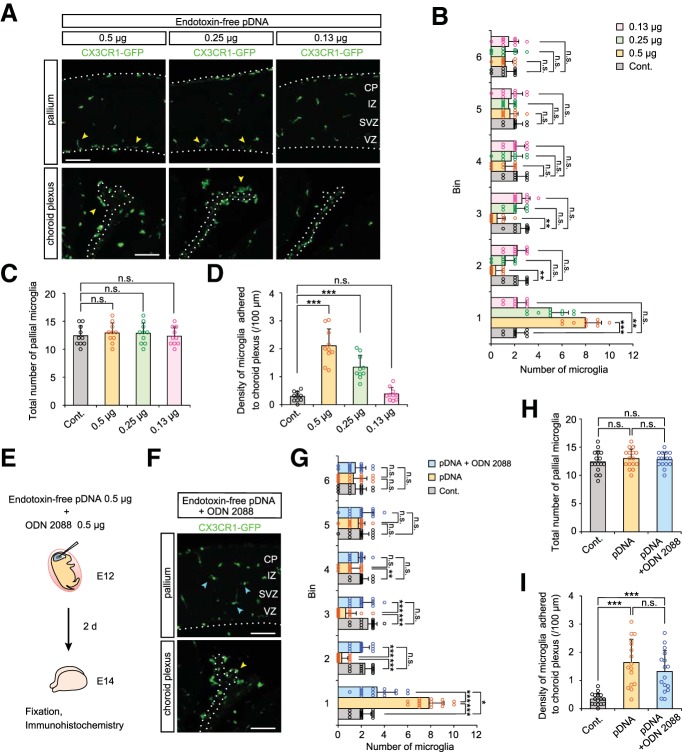
**Plasmid DNA itself elicits microglial response via TLR9. *A***, Immunofluorescence with anti-GFP antibody, showing the distribution of microglia in the pallium and choroid plexus in brains injected with the indicated amount of endotoxin-free plasmid DNA (0.5, 0.25, and 0.13 µg). Yellow arrowheads show microglia accumulated near the apical surface and on the choroid plexus. Scale bar, 100 µm. ***B***, ***C***, Graphs depicting the number of pallial microglia positioned in each 40 µm bin (***B***) and the total number of these cells within 240 µm from the apical surface (***C***). ***D***, Density of microglia adhered to the choroid plexus. For statistical analyses in ***B***–***D***, *n* = 10 samples obtained from five embryos (2 sections, each) were quantified. Two or three littermates per dam were subjected to a series of tests. Data represent mean ± SD. ***p < 0.001, **p < 0.01, *p < 0.05, or n.s., not significant; Steel–Dwass test (Fig. 7-1). ***E***, Experimental design for administration of ODN 2088 together with endotoxin-free plasmid DNA. ***F***, CX3CR1-GFP immunostaining showing microglial distribution in brains injected with endotoxin-free plasmid DNA and ODN 2088 coinjected brains. Yellow arrowhead indicates microglia adhered to the choroid plexus. Cyan arrowheads show microglia which were almost homogenously distributed in the cerebral wall. Scale bar, 100 µm. ***G***, ***H***, Graphs depicting the number of pallial microglia positioned in each bin (***G***) and the total number of these cells within 240 µm from the apical surface (***H***). ***I***, Density of microglia adhered to the choroid plexus. For statistical analyses in ***G***–***I***, *n* = 16 samples obtained from eight embryos (2 sections, each) were quantified. Two or three littermates per dam were subjected to a series *of* tests. Data represent mean ± SD. ***p < 0.001, **p < 0.01, *p < 0.05, or n.s. not significant; Steel–Dwass test (Fig. 7-2).

We tested plasmid DNAs purified using a commercially-sourced endotoxin-free (<0.1 EU/µg plasmid DNA) protocol according to manufacturer's instructions (QIAGEN EndoFree Plasmid Maxi Kit). Similar to ones purified with the QIAGEN Plasmid Maxi Kit, the endotoxin-free DNAs (0.5, 0.25 µg) caused microglial aberrant distribution without an increase of the total number (Fig. 7*A*–*D*; Table 7), but endotoxin-free plasmid DNA did not evoke microglial responses at 0.13 µg, which was >0.03 µg, a dose for DNAs obtained with the QIAGEN Plasmid Maxi Kit which would have contained more endotoxin (Fig. 3). Of note, improvements in the localization of pallial microglia were much more clearly seen when ODN 2088 was coadministrated with endotoxin-free plasmid DNAs (0.5 µg) than used with endotoxin-containing ones (Fig. 7*E*–*H*; Table 7; Fig. 7-1), with a minor microglial infiltration in the choroid plexus (Fig. 7*I*; Fig. 7-2).

**Table 7:** Statistics for Figure 7

**Graph**	**Data structure**	**Type of test**	***p***
Fig. 7*B*, bin 1	Nonparametric	Steel–Dwass	Cont vs 0.5 µg, *p* = 7.4 × 10^−4^; Cont vs 0.25 µg, *p* = 0.0017; Cont vs 0.13 µg, *p* = 0.9880;
Fig. 7*B*, bin 2	Nonparametric	Steel–Dwass	Cont vs 0.5 µg, *p* = 0.0018; Cont vs 0.25 µg, *p* = 0.0557; Cont vs 0.13 µg, *p* = 0.9880;
Fig. 7*B*, bin 3	Nonparametric	Steel–Dwass	Cont vs 0.5 µg, *p* = 0.0018; Cont vs 0.25 µg, *p* = 0.5785; Cont vs 0.13 µg, *p* = 1.0000;
Fig. 7*B*, bin 4	Nonparametric	Steel–Dwass	Cont vs 0.5 µg, *p* = 0.1122; Cont vs 0.25 µg, *p* = 0.6280; Cont vs 0.13 µg, *p* = 0.9880;
Fig. 7*B*, bin 5	Nonparametric	Steel–Dwass	Cont vs 0.5 µg, *p* = 0.5457; Cont vs 0.25 µg, *p* = 0.3241; Cont vs 0.13 µg, *p* = 0.8824;
Fig. 7*B*, bin 6	Nonparametric	Steel–Dwass	Cont vs 0.5 µg, *p* = 0.9691; Cont vs 0.25 µg, *p* = 0.9887; Cont vs 0.13 µg, *p* = 0.9773;
Fig. 7*C*	Nonparametric	Steel–Dwass	Cont vs 0.5 µg, *p* = 0.9011; Cont vs 0.25 µg, *p* = 0.9593; Cont vs 0.13 µg, *p* = 0.9994;
Fig. 7*D*	Nonparametric	Steel–Dwass	Cont vs 0.5 µg, *p* = 9.0 × 10^−4^; Cont vs 0.25 µg, *p* = 9.0 × 10^−4^; Cont vs 0.13 µg, *p* = 0.7593
Fig. 7*G*, bin 1	Nonparametric	Steel–Dwass	Cont vs pDNA, *p* = 3.0 × 10^−6^; pDNA vs. pDNA + ODN 2088, *p* = 5.2 × 10^−6^; Cont vs pDNA + ODN 2088, *p* = 0.0429
Fig. 7*G*, bin 2	Nonparametric	Steel–Dwass	Cont vs pDNA, *p* = 7.0 × 10^−6^; pDNA vs. pDNA + ODN 2088, *p* = 1.1 × 10^−5^; Cont vs pDNA + ODN 2088, *p* = 0.4461
Fig. 7*G*, bin 3	Nonparametric	Steel–Dwass	Cont vs pDNA, *p* = 6.9 × 10^−5^; pDNA vs. pDNA + ODN 2088, *p* = 4.3 × 10^−4^; Cont vs pDNA + ODN 2088, *p* = 0.2716
Fig. 7*G*, bin 4	Nonparametric	Steel–Dwass	Cont vs pDNA, *p* = 0.0047; pDNA vs. pDNA + ODN 2088, *p* = 0.0900; Cont vs pDNA + ODN 2088, *p* = 0.2895
Fig. 7*G*, bin 5	Nonparametric	Steel–Dwass	Cont vs pDNA, *p* = 0.5717; pDNA vs. pDNA + ODN 2088, *p* = 0.3952; Cont vs pDNA + ODN 2088, *p* = 0.8520
Fig. 7*G*, bin 6	Nonparametric	Steel–Dwass	Cont vs pDNA, *p* = 0.9469; pDNA vs. pDNA + ODN 2088, *p* = 0.9472; Cont vs pDNA + ODN 2088, *p* = 0.9965
Fig. 7*H*	Nonparametric	Steel–Dwass	Cont vs pDNA, *p* = 0.6872; pDNA vs. pDNA + ODN 2088, *p* = 0.9550; Cont vs pDNA + ODN 2088, *p* = 0.7672
Fig. 7*I*	Nonparametric	Steel–Dwass	Cont vs pDNA, *p* = 2.6 × 10^−5^; pDNA vs. pDNA + ODN 2088, *p* = 0.5896; Cont vs pDNA + ODN 2088, *p* = 9.2 × 10^−5^

10.1523/ENEURO.0312-18.2018.f7-1 Figure 7-1. Graph depicting the number of pallial microglia positioned in each 40 µm bin comparing six groups: Fig. 5*D* Cont., Fig. 5*D* plasmid DNA, Fig. 5*D* plasmid DNA + ODN 2088, Fig. 7*G* Cont., Fig. 7*G* endotoxin-free plasmid DNA, and Fig. 7*G* endotoxin-free plasmid DNA + ODN 2088. Data represent mean ± SD (Steel–Dwass test). Download Figure 7-1, EPS file.

10.1523/ENEURO.0312-18.2018.f7-2 Figure 7-2. Graph showing density of microglia adhered to the choroid plexus comparing six groups, Fig. 5*E* Cont., Fig. 5*E* plasmid DNA, Fig. 5*E* plasmid DNA + ODN 2088, Fig. 7*I* Cont., Fig. 7*I* endotoxin-free plasmid DNA, and Fig. 7*I* endotoxin-free plasmid DNA + ODN 2088. Data represent mean ± SD (Steel–Dwass test). Download Figure 7-2, EPS file.

Together, these results strongly suggest that although endotoxin can also disturb microglial distribution, plasmid DNA itself is the major inducer of abnormal distribution of the mid-embryonic (E14) cortical microglia through their activation of TLR9.

## Discussion

**Figure 8.**
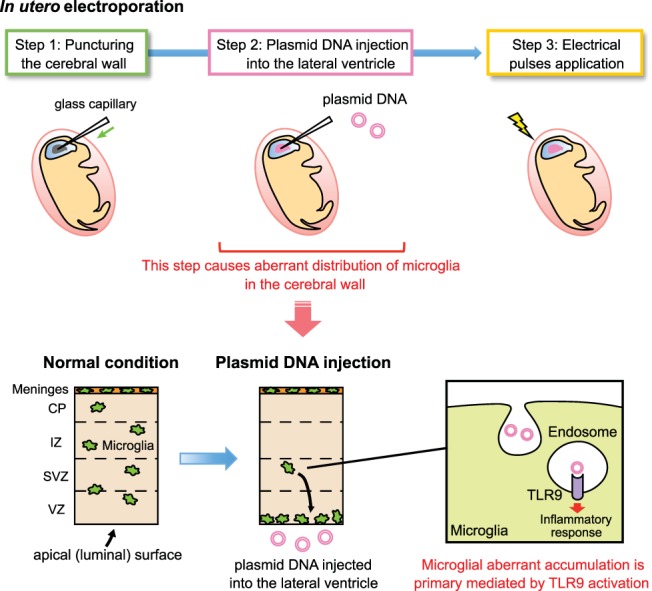
**Schematic summary.** Schematic illustration showing the mechanism underlying the aberrant distribution of microglia in the cerebral wall of IUE-performed brain. The presence of exogenously derived plasmid DNAs induced microglia to accumulate along the apical surface of the cerebral wall and aggregate in the choroid plexus. This effect was independent of capillary needle puncture of the brain wall, or application of electrical pulses. Such microglial response is mediated by activation of TLR9, which is expressed intracellularly in microglia.

Here, we showed that injection of plasmid DNA into the lateral ventricle for IUE induced microglia to accumulate near the luminal surface and aggregate in the choroid plexus, even if electrical pulses were not applied. Notably, this aberrant distribution was triggered through recognition of plasmid DNA by TLR9 expressed in microglia (Fig. 8). Consistent with this, coinjection of a TLR9 antagonist into the ventricle along with plasmid DNA significantly restored the normal, dispersed localization pattern of microglia.

Given that plasmid DNA injection changed the intramural distribution of microglia without changing the total number of microglia per cerebral wall, it is most likely that the observed disappearance of microglia from the IZ and SVZ and their accumulation along the ventricular surface were due to ventricle-directed migration. However, our results do not exclude the possibility that peripheral macrophages infiltrated the embryonic brain, as was very recently shown to occur in response to IUE (Rosin and Kurrasch, 2018). Peripheral macrophage infiltration might underlie the microglial accumulation in the choroid plexus observed in this study. Nevertheless, it is unclear how deeply plasmid DNAs diffuse into the brain wall. We speculate that intra-VZ microglia primarily receive the DNAs and then release certain factors (i.e., cytokines and/or chemokines) that attract other microglia in the IZ or SVZ. Indeed, Rosin and Kurrasch (2018) showed that inflammatory cytokines and chemokines [such as tumor necrosis factor alpha (TNF-α), interleukin-1β (IL-1β), IL-6, MIP-2, RANTES, and MCP-1] were upregulated in embryonic brains following IUE. Although it remains to be determined whether embryonic microglia in the cortex induce these cytokines and chemokines in response to recognition of plasmid DNA, it is understood that TLR9-expressing cells secrete proinflammatory cytokines (such as TNF-α, IL-6, and IL-12) on uptake of CpG motif-containing microbial DNA (Wagner, 2004; Rahmani and Rezaei, 2016). Thus, upregulation of cytokines and chemokines in IUE brains might be induced by TLR9-mediated recognition of plasmid DNA.

TNF-α contributes to the proliferation, differentiation, and survival of neural stem/progenitor cells in the brain (Bernardino et al., 2008; Peng et al., 2008; Lan et al., 2012; Kim et al., 2018). IL-6 promotes differentiation of cortical precursor cells into oligodendrocytes and astrocytes (Bonni et al., 1997; Gruol and Nelson, 1997; Rajan and McKay 1998; Nakanishi et al., 2007; Shigemoto-Mogami et al., 2014), activates adult astrocytes (Campbell et al., 1993), and functions as a neurotrophic and differentiation factor for neurons of the central and peripheral nervous systems (Satoh et al., 1988; Thier et al., 1999; Nakafuku et al., 1992; Murphy et al., 2000; Erta et al., 2012). Therefore, although IUE itself has no effect on apoptosis in neural lineage cells (Zhang et al., 2014; Rosin and Kurrasch, 2018), we cannot exclude the possibility that cytokines produced by microglia expressing TLR9 could modify the physiologic environment in IUE brain.

We showed that exposure to as little as 25 pg of intraventricular LPS (a smaller amount than that contained in plasmid DNA solutions purified with the QIAGEN plasmid Maxi Kit) could attract microglia toward the apical surface. Importantly, although ODN 2088 coadministration coupled with endotoxin-free plasmid DNAs restored microglial aberrant distribution, it did not completely inhibit microglial aggregation in the choroid plexus, indicating that other molecular mechanisms might function for sensing plasmid DNAs. Previous studies revealed that double-stranded DNA complexed with cationic liposomes can induce type I interferon independently of CpG motifs in mouse embryonic fibroblasts and HEK293 cells, which do not express TLR9 (Ishii et al., 2006; Shirota et al., 2006). Recently, Takaoka et al. (2007) reported a cytoplasmic DNA sensor, DNA-dependent activator of IFN-regulatory factors (DAI), that recognizes double-stranded DNA and activates innate immune responses independently of TLR9. Further studies are required to elucidate whether a TLR9-independent immune response to plasmid DNA occurs in microglia.

In summary, intraventricular plasmid DNA injection, a procedure essential for standard IUE techniques, can induce abnormal microglial behaviors in developing cortical walls. These abnormalities can be partly prevented by application of the TLR9 antagonist ODN2088. Overall, our findings emphasize that studies of embryonic microglia following IUE should be interpreted with caution.

## Synthesis

Reviewing Editor: Orly Reiner, Weizmann Institute of Science Decisions are customarily a result of the Reviewing Editor and the peer reviewers coming together and discussing their recommendations until a consensus is reached. When revisions are invited, a fact-based synthesis statement explaining their decision and outlining what is needed to prepare a revision will be listed below. Note: If this manuscript was transferred from JNeurosci and a decision was made to accept the manuscript without peer review, a brief statement to this effect will instead be what is listed below.

This manuscript advances the field with regards to in utero electroporation, an important and widely used method of gene delivery for the study of embryonic cortical cells during fetal development.

This manuscript advances the field by providing a quantitative study of the effects of exogenously derived plasmid DNA on the intramural distribution of microglia, marked by CXCR1-GFP transgene expression. This is an important finding, as a recent study reported infiltrating macrophages as the source of microglia following electroporation. Moreover, the titration of DNA concentrations and the apparent restoration of microglia distribution through pharmacological blockade of TLR9 (upon administration of ODN2088) is noteworthy, and offers a clear direction of further research.

The focus of this study is to understand the impact of in utero electroporation of plasmid DNA on the distribution of microglia, marked by CX3CR1-GFP transgene expression, within the developing forebrain. The authors first performed in utero electroporation and discovered an unexpected, intramural distribution of CX3CR1-GFP+ microglia, most notably within the luminal wall. The total numbers of GFP+ cells within the field of view for quantification was not significantly different. This was followed by a careful study to determine that the presence of exogenously sourced plasmid DNA, rather than glass capillary needle puncture or application of electrical pulses was responsible for the altered distribution of resident microglia, although the authors are pragmatic to conclude that they cannot rule out the possibility that infiltrating macrophages may also explain their observation. Next, the authors performed a titration study to find a threshold of plasmid DNA which triggers intramural microglial positioning within the embryonic neocortex, and dose-dependent accumulation of microglia within the choroid plexus. Finally, the authors provide evidence that TLR9 is highly enriched in CX3CR1-GFP expressing microglia of the embryonic pallium, and that the intramural changes in microglial distribution upon plasmid electroporation is significantly impeded by blockade of TLR9 by application of the oligonucleotide ODN2088.

Overall, these findings are highly significant and will be of great interest to developmental neurobiologists. This research is timely because in utero electroporation is a widely used approach to manipulate gene expression in embryonic brain cells, and the influence on the resident as well as infiltrating microglia of the embryonic neocortex remains to be better understood. The study is carefully conducted, beautifully illustrated, and the results convincing. The conclusions are sound, and the Discussion is very well balanced.

Several issues need to be clarified:

Major issues:

1. In the study of the effects of ODN2088 co-treatment with plasmid DNA electroporation (Fig. 5), the authors should compare with control treatment to determine the extent of ODN restoration of intramural distribution of GFP+ cells, as well as choroid accumulation of GFP+ cells. This is particularly relevant to the authors description that microglia were “...almost normally distributed..” (Fig. 5G) (Line 273), as well as in the Discussion in which it is stated in Lines 286-287 that “...co-injection of TLR9 antagonist into the ventricle along with plasmid DNA restored the normal, dispersed, localization pattern of microglia”. Is the intramural distribution profile for pDNA+ODN2088 not significantly different to control profile?

2. Which Bin includes the surface of the ventricle? Please make sure this is readily apparent to the reader. If Bin 6 includes both microglia within the VZ but near the ventricle, and microglia on the surface of the ventricle this should be made clear to the reader as these may represent distinct cells or distinct reactions to the procedure.

3. Please indicate the approximate location of injection into the E12.5 mouse and report in the methods and results where the coronal sections for analysis were taken. This is important in the context of how local microglia react to a nearby injection versus how they react to presence of DNA in the ventricle.

4. In the methods and results it is vitally important to list the number of embryos per litter that were injected, and the number of pregnant mice that were used for every experiment. These numbers are crucial. The methods lists “n {greater than or equal to} 4” under statistics. But the authors must include more info as mentioned: number of embryos used for analysis and the number of pregnant dams that the embryos were taken from.

5. Microglia are present on the surface of the ventricle in normally developing prenatal cortex in other species. If there are truly none in mice, fine, but report the number of embryos analyzed and how many litters they were taken from. There can be animal to animal variability and litter to litter variability, particularly at early stages of development. The reader should get a clear picture of how often the results occur: Does DNA injection always produce altered distribution? Do control injections never produce altered distribution? These data are necessary.

Minor issues:

1. Lines 12-14 of the ABSTRACT reads “...luminal entry, intra-ventricular administration of plasmid DNAs (i.e., not involving the puncture of the brain wall by capillary needle or application of electrical pulses) induced microglia to accumulate on the luminal surface and and aggregate in the choroid plexus.”

I believe that this sentence is a little bit misleading, as the delivery of all agents (TE buffer, ODN2088, plasmid DNA) involve the puncture of the brain wall. Respectfully, could the authors please consider revising this sentence, perhaps to the following lines of text which I have provided in square brackets [ ]:

“...luminal entry, [the presence of exogenously derived plasmid DNAs] induced microglia to accumulate on the luminal surface and aggregate in the choroid plexus. [This effect was independent of capillary needle puncture of the brain wall, or application of electrical pulses]. [The] microglial response occurred at DNA concentrations.....”

2. In Line 90-91 of page 3 when the authors mention “...and potential technical solutions for this artificial phenomenon”, it might be better to replace this expression to read “...and point to a potential molecular mechanism for this phenomenon”.

3. Could the authors please consider re-wording line 220 of page 8 describing “...artificial presence of plasmid DNAs...” to “...the presence of exogenously sourced plasmid DNAs”, or the like.

4. In Lines 230-232, the authors discuss findings with regards to microglial distribution in response to different concentrations of injected plasmid. They should refer to Fig. 3A,B on Line 232 (instead of Fig. 3A), and refer to Fig. 3C on Line 233 (instead of Fig. 3B).

5. In Fig.5A, it is difficult to determine from the graph if CX3CR1-negative cells express TLR9 at all - there is no visible column or error bar, which could suggest that their expression is zero relative units. Therefore, the authors' interpretation that that the GFP-positive microglia expressed markedly higher levels is difficult to substantiate. Please consider revising the sentence, or providing a value for CX3CR1-GFP negative cells and positive cells in relative units; or a numerical fold-difference value (e.g. 500-fold higher level of TLR9 compared with -negative cells?). This would clarify the issue and validate the authors' statement in Line 261 that “microglia express markedly higher levels...”.

6. Related to the data on GFP+ cell distribution, could the authors please provide a schematic or details for what each Bin (1-6) represents? For example, does Bin6 encompass cells in the pail surface as well? A description in the Methods section would be sufficient.

7. For Fig. 2, quantification of GFP+ cell distribution across conditions would be very helpful here. Please provide the graphs and statistical validation of observations, as well as data on the numbers of GFP+ cells in the cortex and choroid plexus as well.

8. Could the authors please comment on the distribution of CX3CR1-GFP+ microglia in the pial surface? Based on the images provided in the manuscript data, there appears to be no effect on the distribution of GFP+ cells in the pial surface - is this correct? Related to this, GFP+ cells within the pial surface are not represented in the schematic in Fig 6, notably in images for both “Normal Condition” and “Plasmid DNA injection”.

9. An additional control would be the delivery of a scrambled ODN to show that there is a specific blockade of TLR9 signalling which influences microglial infiltration.

## Author Response

October 12, 2018

Dear Editors and Reviewers,

Thank you so much for giving us the opportunity to make revisions. Before addressing the issues raised by the previous review, we would like to add a new data about endotoxin to this revised manuscript.

It is important to note that the plasmid DNA purified with the QIAGEN Plasmid Maxi kit (catalog number 12163), which we routinely use for in utero electroporation (IUE), contains a tiny level of bacterial endotoxin, known as lipopolysaccharide (LPS). We newly found that intraventricularly injected LPS led to microglial accumulation along the apical surface of the cerebral wall and in the choroid plexus. Thus, we separately tested whether endotoxin-free plasmid DNA purified with QIAGEN Endofree Plasmid Kit (catalog number 12362) would disturb microglial distribution, and found that plasmid DNA itself induced the abnormal localization of microglia. Of note, ODN 2088 treatment coupled with endotoxin-free plasmid DNAs improved much more than using endotoxin-containing ones, suggesting that LPS contained in plasmid DNA solution would augment microglial responses.

Accordingly, the new result section was appended to the original version, numbered as “5”. The section title is:

5. Endotoxins, if contained in plasmid DNA solution, trigger microglial aberrant accumulation (page 11-12).

In this section, we described the inspections for microglial responses to intraventricularly administered LPS (new Fig. 6), and ones to endotoxin-free plasmid DNAs and the recovering effect of ODN 2088 for microglial aberrant distribution (new Fig. 7). Figure legends for them are shown in page 22-23. The text regarding these contents were shown in red letters.

In accordance with the insertion of new figures, we renumbered the original Fig.6 (showing the schematic summary) as Fig. 8. Changes to the manuscript are highlighted in yellow.

Line 378 of page 13.

Here, we showed that injection of plasmid DNA into the lateral ventricle for IUE induced microglia to accumulate near the luminal surface and aggregate in the choroid plexus, even if electrical pulses were not applied. Notably, this aberrant distribution was triggered through recognition of plasmid DNA by TLR9 expressed in microglia (Fig. 8).

Based on these new findings, a description for our interpretation has been changed in discussion section as follows.

Line 408-413 of page 14.

We showed that a very small amount of intraventricular LPS (equivalent to its amount contained in plasmid DNA solutions purified with the QIAGEN plasmid Maxi Kit) could attract microglia towards the apical surface. Importantly, although ODN 2088 co-administration coupled with endotoxin-free plasmid DNAs restored microglial aberrant distribution, it did not completely inhibit microglial aggregation in the choroid plexus, indicating that other molecular mechanisms might function for sensing plasmid DNAs. Previous studies revealed that double-stranded DNA complexed with cationic liposomes can induce type I interferon independently of CpG motifs in mouse embryonic fibroblasts and HEK293 cells, which do not express TLR9 (Ishii et al., 2006; Shirota et al., 2006).

Then, we would like to move on the revised contents based on the given comments. We sincerely thank for insightful suggestions and have carefully reviewed all of them. Our responses are given in point-by-point manner below.

Major issues:

1. In the study of the effects of ODN2088 co-treatment with plasmid DNA electroporation (Fig. 5), the authors should compare with control treatment to determine the extent of ODN restoration of intramural distribution of GFP+ cells, as well as choroid accumulation of GFP+ cells. This is particularly relevant to the authors description that microglia were “...almost normally distributed.” (Fig. 5G) (Line 273), as well as in the Discussion in which it is stated in Lines 286-287 that “...co-injection of TLR9 antagonist into the ventricle along with plasmid DNA restored the normal, dispersed, localization pattern of microglia”. Is the intramural distribution profile for pDNA+ODN2088 not significantly different to control profile?

I appreciate this helpful suggestion. We newly added the data of the quantification of control (intact) and ODN 2088-treated (without plasmid DNAs) brains, and compared plasmid DNA + ODN 2088-co-injected brains and these groups (Fig. 5D-F). In plasmid DNAs (endotoxin-containing) and ODN 2088-co-injected brains, the number of microglia in bin 1 was significantly smaller than that of plasmid DNA-treated brains, but it did not decrease as much as control or only ODN 2088-treated brains. As noted in the previous page, ODN 2088 treatment, if we used endotoxin-free plasmid DNAs, improved much more than using endotoxin-containing ones (Fig. 7), suggesting that LPS contamination in plasmid DNA solution may be the cause that ODN 2088 treatment could not completely restore microglial aberrant distribution (Fig. 5).

In the result section 4, we concluded for this time that ODN 2088 treatment could not completely restore microglial distribution when coupled with endotoxin-containing plasmid DNAs. To make it easier to understand, we revised the manuscript as follows.

Line 301-305 of page 10

ODN 2088 treatment partially restored the number of microglia localized in the SVZ/IZ and significantly reduced their accumulation into the apical surface, although it did not entirely rescue abnormal distribution (the number of microglia in bin 1 was still higher than control [non-treated] or only ODN 2088-treated brains) (Fig. 5C, D; Table 5). In addition, microglial infiltration in the choroid plexus was significantly reduced in ODN 2088-treated brain but still greater than control groups (Fig. 5E).

We also revised Fig.5 legend as follows.

Line 660-661 of page 22

(D, E) Graphs indicate the number of CX3CR1-GFP+ cells in each 40-µm bin of the pallium (D) and density of microglia directly adhered to the choroid plexus (E), comparing control, only ODN 2088-treated, plasmid DNA-injected, and plasmid DNA + ODN 2088-co-injected brains. (F) Graph showing the total number of pallial microglia within 240 µm from the apical surface.

2. Which Bin includes the surface of the ventricle? Please make sure this is readily apparent to the reader. If Bin 6 includes both microglia within the VZ but near the ventricle, and microglia on the surface of the ventricle this should be made clear to the reader as these may represent distinct cells or distinct reactions to the procedure.

I appreciate this valuable comment. To make it clear, we added the new figure showing which bin corresponds to the region of the cerebral wall (Fig. 1D) and described the criteria for quantification in the method section. We counted the number of microglia of which somas are inside the VZ, and did not count those positioned outside the cerebral wall (such as ones just attached to the apical surface). We analyzed the all data in a unified manner.

We revised as follows.

Line 147-150 of page 5

Immunohistochemistry

Immunohistochemistry was performed as described previously (Okamoto et al., 2013). Brains were fixed in 4% PFA, immersed in 20% sucrose, and frozen-sections (16 µm thickness) were cut. Sections were treated with the following primary antibodies: rat anti-GFP (1:500, Nacalai tesque, Kyoto, Japan) and rabbit anti-RFP (1:500, MBL, Aichi, Japan). After washes, sections were treated with secondary antibodies conjugated to Alexa Fluor 488 or Alexa Fluor 546 (1:400, Molecular Probes, Eugene, OR, USA) and imaged on a BX60 fluorescence microscope (Olympus, Tokyo, Japan) or FV1000 confocal microscope (Olympus). The cerebral wall was divided into 6 bins (40-µm) and numbered from inside (bin 1-6). We counted the number of microglia of which somata were within the VZ (including ones along the apical surface) but did not count microglia whose somata were completely in the ventricular lumen even though they partly attached to the apical surface.

Line 601-603 of page 20. (legend for Fig. 1)

(D) Bin definition for immunohistochemical analyses is shown. Each section in the cerebral wall was numbered from the ventricle side (bin 1-6, 40-µm each).

Further, we noted the specific bin number information in the result.

Line 230-232 of page 8 (Results, section 1)

In brains subjected to IUE (hereafter, IUE brains), however, microglia were extremely scarce in both the SVZ and IZ (bins 2-4) and aberrantly accumulated along the apical surface (within 40 µm from the apical surface: bin 1) (Fig. 1C-E; Table 1), ...

3. Please indicate the approximate location of injection into the E12.5 mouse and report in the methods and results where the coronal sections for analysis were taken. This is important in the context of how local microglia react to a nearby injection versus how they react to presence of DNA in the ventricle.

Thanks for this suggestion. We added the location information for the injection into the E12.5 mouse brain and for immunohistochemical analyses in E14.5 in the method and result as follows.

Line 110-113 of page 4 (Methods: Plasmid DNA and LPS injection into the lateral ventricle)

One microliter of plasmid DNA solution was injected into the lateral ventricle of the right hemisphere of E12 mouse brain. The final concentration of plasmid DNA ranged 0.03-0.5 µg/µl, as indicated. After two days, the number and distribution pattern of microglia were quantified in the lateral part of the cerebral wall and choroid plexus (right hemisphere) (Fig. 1B).

Line 222-224 of page 8 (Results, section 1)

Briefly, plasmid DNA (pEFX2-Lyn-mCherry) was injected into the lateral ventricle of the right hemisphere of E12 mouse brain using a glass capillary needle, followed by electrical pulses across the embryo's head (Fig. 1A). Surprisingly, immunohistochemical inspections 2 days later revealed that the distribution patterns of CX3CR1+ microglia in the pallium and choroid plexus were abnormal (Fig. 1B, C).

4. In the methods and results it is vitally important to list the number of embryos per litter that were injected, and the number of pregnant mice that were used for every experiment. These numbers are crucial. The methods lists “n {greater than or equal to} 4” under statistics. But the authors must include more info as mentioned: number of embryos used for analysis and the number of pregnant dams that the embryos were taken from.

I appreciate this valuable comment. We added detail information about samples for quantification in each figure legend.

Line 606-607 of page 20 (legend for Fig. 1)

For statistical analyses, n = 10 samples obtained from 5 embryos (two sections, each) were quantified. One or two littermates per dam were subjected to a series of tests.

Line 621-623 of page 21 (legend for Fig. 2)

For statistical analyses, n = 10 samples obtained from 5 embryos (two sections, each) were quantified. One or two littermates per dam were subjected to a series of tests.

Line 632-634 of page 21 (legend for Fig. 3)

For statistical analyses, n = 10 samples obtained from 5 embryos (two sections, each) were quantified. One or two littermates per dam were subjected to a series of tests.

Line 643-645 of page 21 (legend for Fig. 4)

For statistical analyses, n = 10 samples obtained from 5 embryos (two sections, each) were quantified. Two or three littermates per dam were subjected to a series of tests.

Line 665-666 of page 22 (legend for Fig. 5)

For statistical analyses, n = 16 samples obtained from 8 embryos (two sections, each) were quantified. Two or three littermates per dam were subjected to a series of tests.

Line 680-682 of page 22 (legend for Fig. 6)

For statistical analyses, n = 10 samples obtained from 5 embryos (two sections, each) were quantified. Two or three littermates per dam were subjected to a series of tests.

Line 691-693 of page 23 (legend for Fig. 7)

For statistical analyses, n = 10 samples obtained from 5 embryos (two sections, each) were quantified. Two or three littermates per dam were subjected to a series of tests.

Line 700-702 of page 23 (legend for Fig. 7)

For statistical analyses, n = 16 samples obtained from 8 embryos (two sections, each) were quantified. Two or three littermates per dam were subjected to a series of tests.

Accordingly, we added a new sentence in the method section as follows.

Line 189 of page 7

Statistical analysis

Quantitative data are presented as means {plus minus} S.D. from representative experiments. Statistical analyses were performed using the Mann-Whitney U test for two-group comparisons or using Steel-Dwass test for multiple comparisons. P &lt; 0.05 was considered significant. P-values in every figure are separately listed in tables (Table 1-7). Individual values were plotted as open circles in bar graphs. The number of samples examined in each analysis is shown in the figure legends.

5. Microglia are present on the surface of the ventricle in normally developing prenatal cortex in other species. If there are truly none in mice, fine, but report the number of embryos analyzed and how many litters they were taken from. There can be animal to animal variability and litter to litter variability, particularly at early stages of development. The reader should get a clear picture of how often the results occur: Does DNA injection always produce altered distribution? Do control injections never produce altered distribution? These data are necessary.

I appreciate this suggestion. As mentioned in “major issue 2”, we only counted the number of microglia present within the VZ of the cerebral wall. We found some cortical microglia on the apical surface in normal conditions, but it seemed a rare case in mouse brains. (On the other hand, we did not evaluate luminal microglia just attached to the ventricular surface because numerical variations were large depending on the scale of microglial aggregation in the ventricular lumen.)

To enable readers to understand the variability between animals at a glance, we provided the individual values in all figures (shown in dot plots in every bar graph). As answered in the previous topic, we also described detail information about analyzed samples, such as how many embryos were tested and how many pregnant dams were used. As you will find out from the dot plots, microglial aberrant distribution was confirmed in all plasmid DNA-injected (0.5 µg) cases, although the recovery effect by ODN 2088 treatment seemed to be relatively variable depending on individual animals. In addition, as far as we analyzed, microglia never altered their distribution of the cerebral wall under control injections (Tris-EDTA solution).

In accordance with the addition of individual values in results, we revised the manuscript as follows.

Line 187-189 of page 7

Statistical analysis

Quantitative data are presented as means {plus minus} S.D. from representative experiments. Statistical analyses were performed using the Mann-Whitney U test for two-group comparisons or using Steel-Dwass test for multiple comparisons. P &lt; 0.05 was considered significant. P-values in every figure are separately listed in tables (Table 1-7). Individual values were plotted as open circles in bar graphs. The number of samples examined in each analysis is shown in the figure legends.

Minor issues:

1. Lines 12-14 of the ABSTRACT reads “...luminal entry, intra-ventricular administration of plasmid DNAs (i.e., not involving the puncture of the brain wall by capillary needle or application of electrical pulses) induced microglia to accumulate on the luminal surface and and aggregate in the choroid plexus.”

I believe that this sentence is a little bit misleading, as the delivery of all agents (TE buffer, ODN2088, plasmid DNA) involve the puncture of the brain wall. Respectfully, could the authors please consider revising this sentence, perhaps to the following lines of text which I have provided in square brackets [ ]:

“...luminal entry, [the presence of exogenously derived plasmid DNAs] induced microglia to accumulate on the luminal surface and aggregate in the choroid plexus. [This effect was independent of capillary needle puncture of the brain wall, or application of electrical pulses]. [The] microglial response occurred at DNA concentrations.....”

Thanks for this comment. We revised the manuscript as follows.

Line 11-15 of page 1

However, we unexpectedly faced a technical problem: although microglia are normally distributed homogeneously throughout the mid-embryonic cortical wall with only limited luminal entry, the intraventricular presence of exogenously derived plasmid DNAs induced microglia to accumulate along the apical surface of the cortex and aggregate in the choroid plexus. This effect was independent of capillary needle puncture of the brain wall or application of electrical pulses. The microglial response occurred at plasmid DNA concentrations lower than those usually used for IUE,

2. In Line 90-91 of page 3 when the authors mention “...and potential technical solutions for this artificial phenomenon”, it might be better to replace this expression to read “...and point to a potential molecular mechanism for this phenomenon”.

Thanks for the suggestion. We revised the text as follows.

Line 90-91 of page 3

In this study, we investigated the causes of abnormal microglial distribution and point to a potential molecular mechanism for this phenomenon.

3. Could the authors please consider re-wording line 220 of page 8 describing “...artificial presence of plasmid DNAs...” to “...the presence of exogenously sourced plasmid DNAs”, or the like.

Thanks for the suggestion. We revised the text as follows.

Line 253-254 of page 9

These results strongly suggest that the presence of exogenously sourced plasmid DNAs in embryonic mouse ventricle caused abnormal microglial distribution.

4. In Lines 230-232, the authors discuss findings with regards to microglial distribution in response to different concentrations of injected plasmid. They should refer to Fig. 3A,B on Line 232 (instead of Fig. 3A), and refer to Fig. 3C on Line 233 (instead of Fig. 3B).

Thanks for the comment. We revised the manuscript as follows.

Line 264-265 of page 9

After 2 days (E14), microglial accumulation near the ventricle was still observed in brains injected with 0.25 µg, 0.13 µg, or 0.06 µg plasmid DNA (Fig. 3A, B; Table 3), with no increase of the total number of pallial microglia (Fig. 3C).

5. In Fig.5A, it is difficult to determine from the graph if CX3CR1-negative cells express TLR9 at all - there is no visible column or error bar, which could suggest that their expression is zero relative units. Therefore, the authors' interpretation that that the GFP-positive microglia expressed markedly higher levels is difficult to substantiate. Please consider revising the sentence, or providing a value for CX3CR1-GFP negative cells and positive cells in relative units; or a numerical fold-difference value (e.g. 500-fold higher level of TLR9 compared with -negative cells?). This would clarify the issue and validate the authors' statement in Line 261 that “microglia express markedly higher levels...”.

We'd like to thank for this helpful suggestion. We provided a numerical fold-difference value for CX3CR1-GFP negative cells and positive cells.

We revised text as follows:

Line 295-297 of page 10

To determine whether embryonic microglia express TLR9, we performed real-time quantitative PCR on CX3CR1-GFP+ microglia and CX3CR1-GFP- cells (most of which are of the neural lineage) isolated by cell sorting from the cortical wall of E14 CX3CR1-GFP mice. CX3CR1+ microglia expressed 529-fold higher level of TLR9 compared with CX3CR1- cells (p = 0.0286, Mann-Whitney U test) (Fig. 5A).

6. Related to the data on GFP+ cell distribution, could the authors please provide a schematic or details for what each Bin (1-6) represents? For example, does Bin6 encompass cells in the pail surface as well? A description in the Methods section would be sufficient.

I appreciate the valuable suggestion. As mentioned above (major point 2), we newly added a figure representing which bin corresponds to the region of the cerebral wall (Fig. 1D). In this definition, Bin 6 does not contain microglia in the pial surface.

7. For Fig. 2, quantification of GFP+ cell distribution across conditions would be very helpful here. Please provide the graphs and statistical validation of observations, as well as data on the numbers of GFP+ cells in the cortex and choroid plexus as well.

Thanks for this comment. The graphs of statistical analyses were newly provided in Fig. 2 panel. In accordance with this, we revised text as follows.

Line 247 of page 8

When the cerebral wall was only punctured with a glass capillary needle, but no solution was injected, microglia were still distributed homogenously throughout the cortex, as in control (non-treated) brains (Fig. 2A-D, Table 2).

Line 253 of page 9

In another control group injected with Tris-EDTA solution (10 mM Tris-HCl, 1 mM EDTA, pH 8.0) alone, microglia showed normal distribution pattern in the cerebral wall and did not aggregate in the choroid plexus (Fig. 2A-D).

Legend for figure 2 has been also revised.

Line 619-621 of page 20

Figure 2 Plasmid DNA injection into the ventricle, without electrical pulses, results in abnormal microglial distribution

(A) Representative immunostaining for CX3CR1-GFP in the pallium and choroid plexus of mouse brains subjected to puncture with a glass capillary needle, injection of plasmid DNA (shown as pDNA) into the lateral ventricle, electrical pulses, or Tris-EDTA solution (10 mM Tris-HCl, 1 mM EDTA, pH 8.0) alone without plasmid DNA. Yellow arrowheads indicate microglia accumulated near the apical surface of the cerebral wall or adhered to the choroid plexus. Broken lines show the apical surface of the pallium in the upper panel and the choroid plexus in the lower panel. Scale bar, 100 µm. (B, C) Graphs depicting the number of microglia positioned in each 40-µm bin (B) and the total number of these cells within 240 µm from the apical surface (C) in brains that were subjected to each procedure. (D) Density of microglia adhered to the choroid plexus. For statistical analyses, n = 10 samples obtained from 5 embryos (two sections, each) were quantified. One or two littermates per dam were subjected to a series of tests. Data represent means {plus minus} S.D. (***p &lt; 0.001, **p &lt; 0.01, *p &lt; 0.05, or n.s. [not significant]; Steel-Dwass test).

8. Could the authors please comment on the distribution of CX3CR1-GFP+ microglia in the pial surface? Based on the images provided in the manuscript data, there appears to be no effect on the distribution of GFP+ cells in the pial surface - is this correct? Related to this, GFP+ cells within the pial surface are not represented in the schematic in Fig 6, notably in images for both “Normal Condition” and “Plasmid DNA injection”.

I appreciate this helpful suggestion. We additionally investigated whether the number of meningeal microglia may alter in IUE brains. As newly shown in Fig. 1G, we did not detect a significant difference between control or IUE brains.

Accordingly, we revised text as follows.

Line 232-233 of page 8 (Results, section 1)

In brains subjected to IUE (hereafter, IUE brains), however, microglia were extremely scarce in both the SVZ and IZ (bins 3 and 4) and aberrantly accumulated along the apical surface (within 40 µm from the apical surface: bin 1) (Fig. 1C-E), with the total number of microglia in the pallium and meningeal microglia comparable between control (non-IUE) and IUE brains (Fig. 1F, G).

Line 605 of page 20 (legend for Fig. 1)

(E-G) Graphs depicting numbers of microglia in each bin (300-µm width) (E), the total number within 240 µm from the apical surface of the pallium (F), and the number of meningeal microglia (G).

We also changed the schematic illustration depicting microglial localization in the mouse cerebral wall in both images for “Normal Condition” and “Plasmid DNA injection” in Fig. 8 (originally Fig 6, but renumbered in this revised manuscript) (right panel). Microglia in the meninges have been shown.

9. An additional control would be the delivery of a scrambled ODN to show that there is a specific blockade of TLR9 signaling which influences microglial infiltration.

I appreciate this suggestion. Previous report demonstrated that the oligonucleotide (ODN) containing the motif “not C, unmethylated C, G, not G” is the most powerful mitogen for TLR9 activation through the thorough analyses testing various sequences (Krieg et al., 1995). Further, the subsequent study identified the critical bases position before and after “unmethylated C, G” for deciding stimulatory or inhibitory property for TLR9 activation (Stunz et al., 2002). For example, replacing the bases of a stimulatory sequence GCGTTG (ODN 2147) with GCGGGG (ODN 2088) confers inhibitory property for TLR9 activation. Among of the several developed ODNs, it has been reported that ODN 2088 is the best inhibitor. Since previous studies fully tested the stimulatory or inhibitory capacity of various kinds of ODNs, we did not test each of them in this study, but we referred to these papers in the method section as follows.

Line 131-134 of page 5

Administration of TLR9 antagonist together with plasmid DNA

Previous studies tested various oligonucleotides (ODNs) for their stimulatory or inhibitory activities for TLR9 (Krieg et al., 1995; Stunz et al., 2002). Based on the finding demonstrated ODN 2088 is one of the most effective inhibitors, we applied it in our experiments as TLR9 antagonist. The ODN 2088 (5'-TCC TGG CGG GGA AGT-3') was purchased from Invivogen (San Diego, CA, USA). The drug was suspended in endotoxin-free water and dissolved in plasmid DNA solution at a mass ratio of plasmid DNA/ODN 2088 1:1. Plasmid DNA and ODN 2088 were injected into the lateral ventricles of E12 embryos. After 2 days (E14), the brains were perfused with 4% PFA and subjected to immunohistochemistry.

Finally, we summarized p values in all analyses (except for ones of Fig. 4A and Fig.6A) in tables. Tables (Table 1-7) were newly provided in the Word file of the manuscript (page 25-37).

We sincerely thank again the editor and reviewers for giving us a chance to revise this manuscript.

Sincerely,

Yuki Hattori

Yuki Hattori Ph.D.

Department of Anatomy and Cell Biology,

Nagoya University Graduate School of Medicine,

65 Tsurumai, Showa, Nagoya 466-8550, Japan

ha-yuki@med.nagoya-u.ac.jp

tel +81-52-744-2035
